# Supplementary material for: Conditional chemoconnectomics (cCCTomics) as a strategy for efficient and conditional targeting of chemical transmission
Source: eLife. 2024 Apr 30;12:RP91927. doi: 10.7554/eLife.91927 (PMC11060718; doi:10.7554/eLife.91927)
Supplement: Figure 3—source data 3. [file elife-91927-fig3-data3.zip › Figure 3 source data3 part 1.zip!/Figure 3-Source data 3-part1/1067-9-CRISPResso_Report_c33kf3u3.zip!/CRISPResso_on_1067_9/4d.Position_dependent_average_indel_size.pdf]

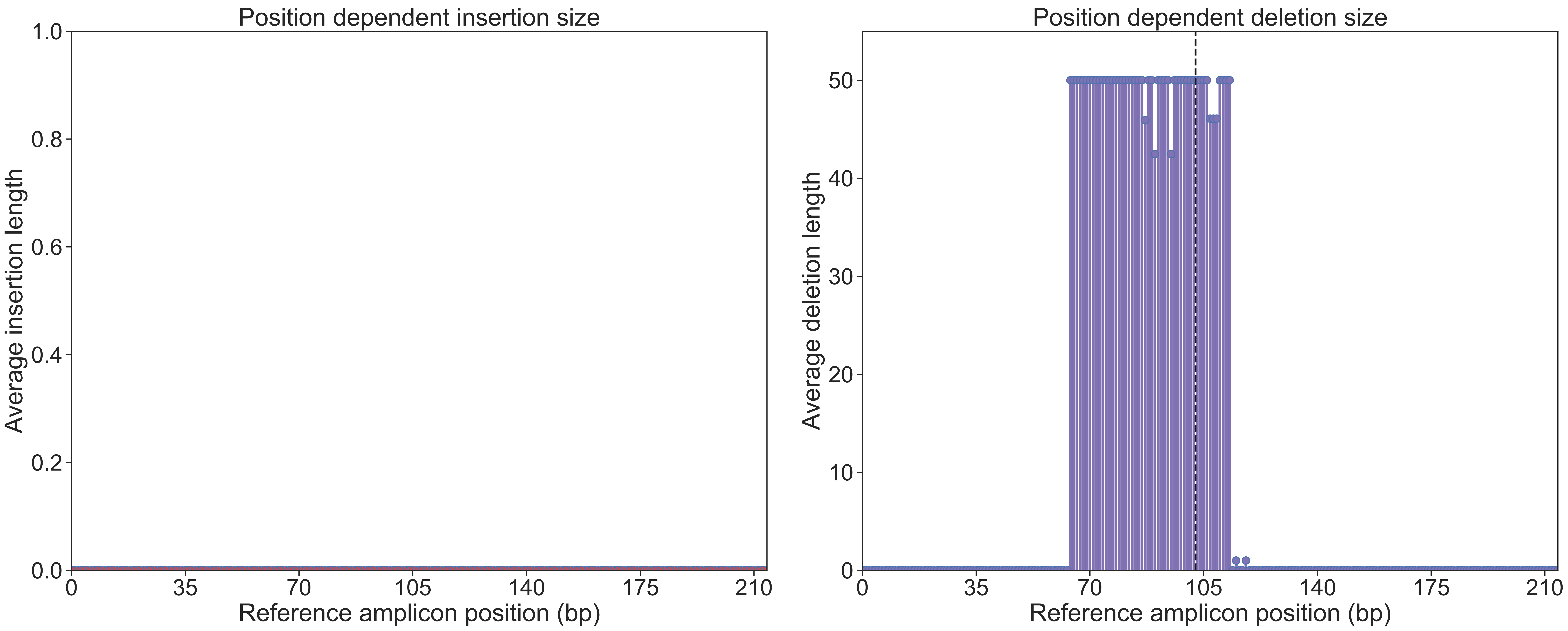
Position dependent insertion size
Average insertion length
1.0
0.8
0.6
0.4
0.2
0.0
0
35
70
105
140
175
210
Reference amplicon position (bp)
Position dependent deletion size
Average deletion length
50
40
30
20
10
0
0
35
70
105
140
175
210
Reference amplicon position (bp)
